# Comparative Evaluation of Autologous Platelet-Rich Plasma and Artificial Tears for Treatment of Chronic Evaporative Dry Eye Patients: A Prospective Interventional Study

**DOI:** 10.7759/cureus.88140

**Published:** 2025-07-17

**Authors:** Pooja Singh, Divya Singh, Amarnath Pandey, Lakkshya Sharma, Sanchita Gupta, Jawahar L Goyal

**Affiliations:** 1 Ophthalmology, School of Medical Sciences and Research, Sharda University, Greater Noida, IND; 2 Transfusion Medicine, School of Medical Sciences and Research, Sharda University, Greater Noida, IND

**Keywords:** artificial tears, autologous platelet rich plasma, autologous serum, corneal fluorescence staining (cfs), dry eye, ocular surface disease index (osdi), schirmer’s test (st), tear breakup time (tbut)

## Abstract

Background: Dry eye is a multifactorial ocular surface disorder. While artificial tears and anti-inflammatory medications are standard treatment, blood derivatives such as platelet-rich plasma (PRP) offer regenerative benefits as well and are gaining popularity in ophthalmology. This study aimed at the comparative evaluation of the clinical outcomes of autologous PRP eye drop and artificial tear eye drop (AT) therapy in the treatment of chronic evaporative dry eye patients with moderate to severe symptoms.

Methods: 100 patients with chronic (≥ 6 months duration) evaporative moderate to severe dry eye disease (DED) in the 18 years to 45 years age group were included in this prospective interventional study. We randomized patients into two equal groups (n = 50 each). One group received autologous PRP eye drops and the other AT drops. The outcome measures evaluated were subjective symptoms (ocular surface disease index (OSDI) score), objective tear film parameters (Oxford corneal fluorescence staining (CFS) score, tear breakup time (TBUT), and Schirmer’s test (ST) score), and best-corrected visual acuity (BCVA) (converted to LogMAR, with improvement defined as a gain of ≥ 1 Snellen line). Within the group and between the group, comparisons were done at the end of 6 months using paired and unpaired student’s t-tests, respectively.

Results: At the end of 6 months, both the treatment groups (PRP and AT) showed significant improvement in OSDI and CFS scores, whereas TBUT and BCVA improved significantly only in the PRP group. No significant changes were observed in ST in either group. The post-treatment, between-the-groups analysis also favored PRP in all the parameters except ST. The PRP group showed significantly better improvements than the AT group in OSDI (MD, i.e., mean difference = -20.9, *t* = -5.12, *P* < 0.0001), CFS score (MD = -0.83, *t* = -5.08, *P* < 0.0001), TBUT (MD = 1.28, *t* = 3.08, *P* = 0.003), and BCVA (MD = -0.025, *t* = -2.15, *P* = 0.034). No significant difference was found in ST (*P* = 0.4). No significant adverse effect was noted throughout the study.

Conclusion: In chronic evaporative DED patients, autologous PRP showed significantly better clinical outcomes than AT and was found to be safe when given for a 6-month duration.

## Introduction

The Ebers Papyrus, 1534 BC, is the very first reference in history to implementing a blood derivative at eye level. In 1975, Ralph et al. pioneered the development of the first portable pump system for delivering plasma to the ocular surfaces of chemical burn patients [1].

A decade later, the academic literature first reported the use of autologous serum (AS) eye drops for the treatment of dry eye disease (DED) associated with Sjögren’s syndrome [2]. While Fox et al. had thus already proposed blood-derived ophthalmic solutions for ocular surface disorders, this therapy gained widespread recognition when, in 1999, Tsubota et al. provided compelling evidence through their two studies regarding the efficacy of AS eye drops in the context of Sjögren’s syndrome-related DED and persistent epithelial defect (PED) [2,3,4]. Their investigations marked the pioneering attempt to systematically ascertain both the therapeutic potential and the safety profile of blood derivatives. Multiple in vivo and in vitro studies in the past have reported the positive impact of hemoderivatives on corneal epithelial cell proliferation, growth, and migration [5].

DED is defined as a multifactorial disorder of tear film homeostasis with four key mechanisms: tear film instability, ocular surface inflammation, hyperosmolarity, and neurosensory abnormality [6]. It is the most common form of the ocular surface disease in clinical practice, and aqueous layer deficiency is a major cause [7]. The currently available treatment modalities are AT substitutes, anti-inflammatory agents, immunosuppressants, and punctal plugs [7,8].

Platelet-rich plasma (PRP), with its rich growth factor content, bacteriostatic, anti-collagenase, and anti-apoptotic properties, along with other key biomechanical effects, is now being increasingly used in ophthalmology [9-11].

This paper looks at the scope of autologous PRP in the Indian context, where the burden of chronic evaporative dry eye is growing due to digitization, pollution, and other lifestyle-related factors. In this study, therefore, we aim for a comparative evaluation of clinical outcomes of autologous PRP eye drops and AT eye drops in the treatment of chronic evaporative dry eye patients with moderate to severe symptoms over a period of 6 months.

## Materials and methods

This was a hospital-based, prospective, randomized interventional study of 6 months' duration. It was conducted on 100 patients with DED from September 2024 to January 2025 at a tertiary eye care center in India. The study was approved by the Institutional Ethics Committee and conducted as per the Declaration of Helsinki. A well-informed and written consent was obtained from all the study participants. Dry eye cases were classified into evaporative and aqueous-deficient types following the guidelines given by Lemp et al. [12]. Disease severity was graded according to the standard Dry Eye Workshop (DEWS) classification scheme. Each participant underwent a standard protocol of ocular examination. Participants were randomized into two equal groups (PRP and AT, n=50 each) using a simple random sampling method with a computer-generated random number table.

Blinding is not implemented in this study. It was not feasible due to visible differences in PRP (yellowish-translucent to opaque) and AT (clear) formulations, the individualized nature of PRP preparation, cold chain requirement, and logistical limitations in maintaining identical packaging and handling.

There was no placebo or control group in this study. Due to ethical concerns in withholding the treatment, we compared PRP with standard AT, reflecting real-world practice.

We included all the patients between 18 years to 45 years of age with symptomatic moderate to severe chronic (of ≥ 6 months duration) evaporative type DED with the ocular surface disease index (OSDI) score of > 40, modified Oxford corneal fluorescence staining (CFS) score ≥ grade 1, tear breakup time (TBUT) of < 10 seconds (s), and Schirmer’s test (ST) score < 10 mm in 5 minutes (Schirmer test I; without anesthesia). Patients were divided into two groups of 50 each: the autologous PRP group and the artificial tear (AT) group.

Any topical medication used formerly by the patients for the treatment of DED was stopped 48 hours before starting the study. The PRP group (50 patients, 100 eyes) was treated with 20% PRP eye drops 4 times a day for 6 months. The AT group (50 patients, 100 eyes) was treated with 0.5% carboxymethyl cellulose eye drops 4 times a day for 6 months. Follow-up was done at 1 week, 2 weeks, 1 month, 3 months, and 6 months. Data were recorded first at the initial visit and then at follow-up visits.

Treatment response was evaluated by the results of the subjective questionnaires and the slit lamp examination. On each follow-up, subjective symptoms and objective signs were evaluated and noted down based on the OSDI score, CFS score, TBUT(s), ST score (mm), and best-corrected visual acuity (BCVA) gain ≥ 1 line on Snellen’s chart and converting it to the LogMAR scale. After the final follow-up, all these parameters were subjected to statistical analysis.

Any uncooperative patient, patients not having refrigeration facility at home, those with aqueous-deficient dry eye disease, diabetic patients and those with systemic causes of DED, patients using certain drugs (e.g., anti-glaucoma medications, beta blockers, oral contraceptive pills, and antihistaminics) or any other medication causing DED as a side effect, patients under 18 years of age and more than 45 years of age, pregnant and lactating females, contact lens wearers, patients with active ocular infection or those who had undergone refractive surgeries, and patients on any other investigational drug trial were all excluded from the study.

Our aim was to maintain a homogeneous study population focused on evaporative DED, which is more common in younger individuals. Patients above 45 were excluded to avoid age-related aqueous deficiency, and getting consent for the interventional study from the parents of those under 18 was an issue.

Process of autologous platelet-rich plasma formation and storage

**Figure 1:**
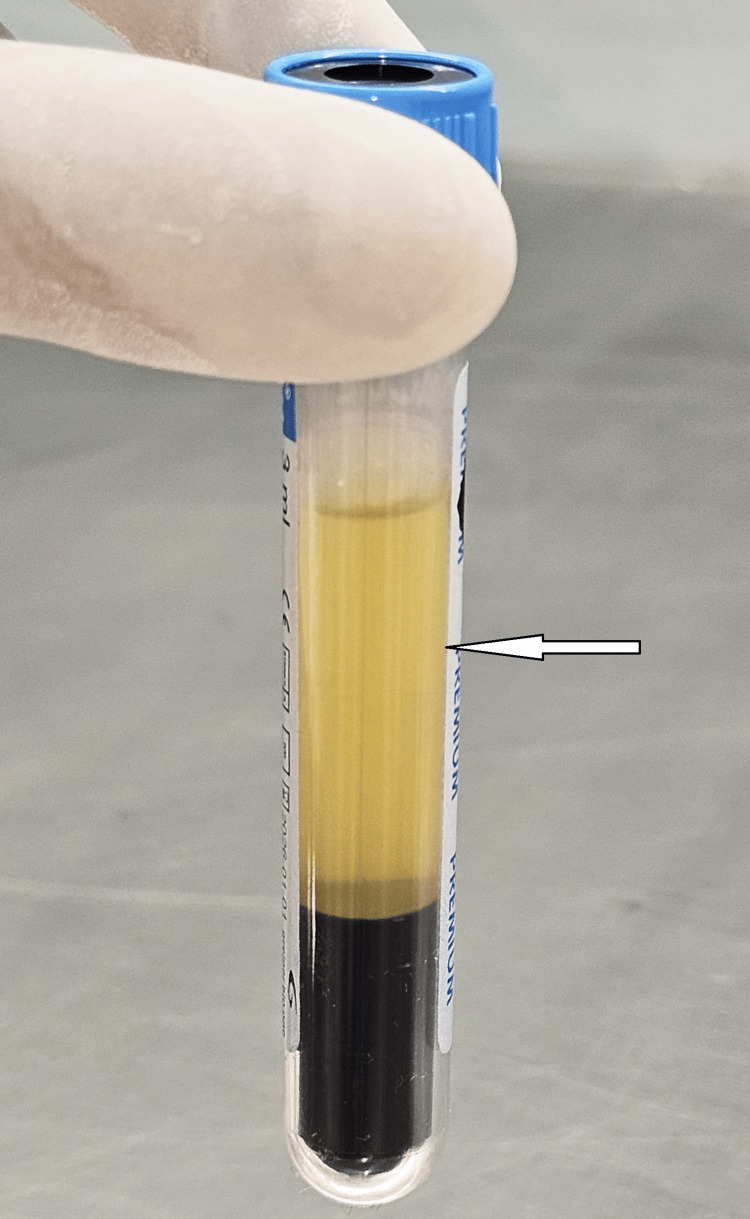
Centrifuged platelet-rich plasma preparation “Arrow” indicates PRP part (PRP: platelet-rich plasma).

**Figure 2:**
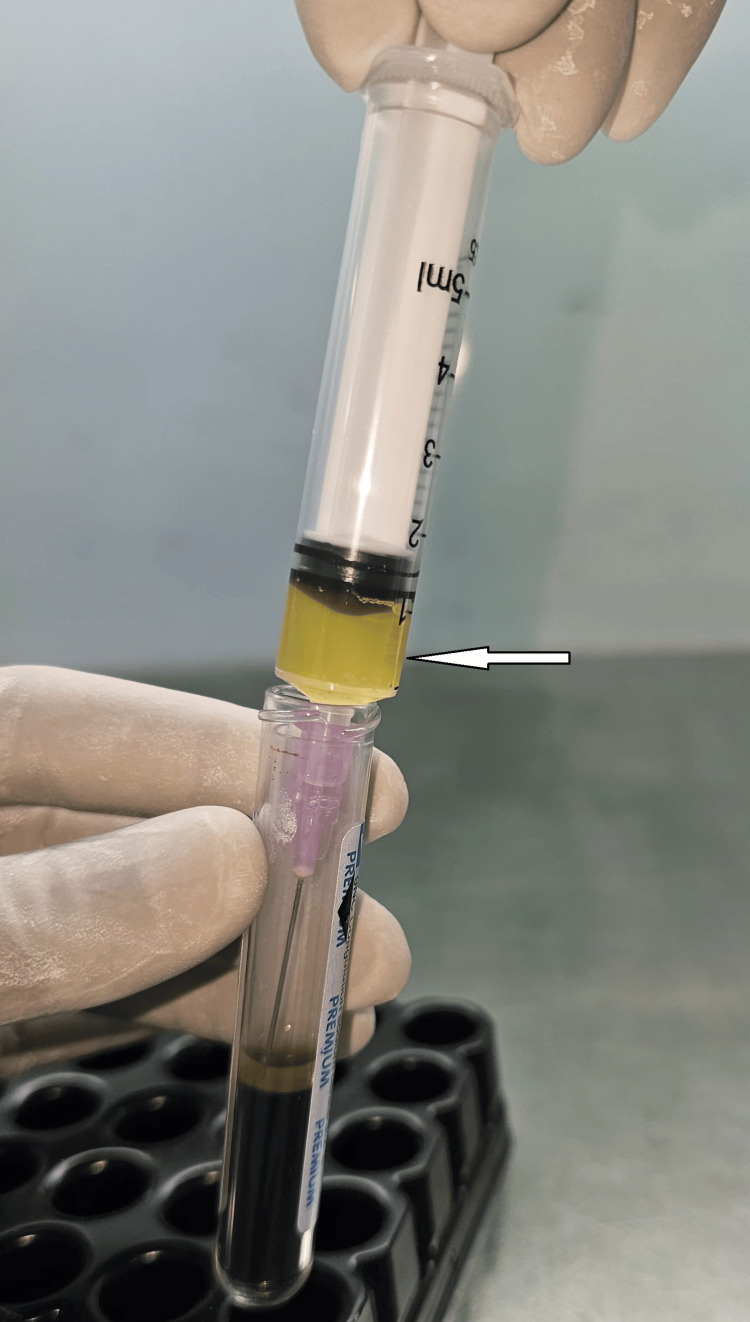
Aspirated PRP aliquot before transfer to sterile eye-drop bottles “Arrow” indicates PRP part (PRP: platelet-rich plasma).

**Figure 3:**
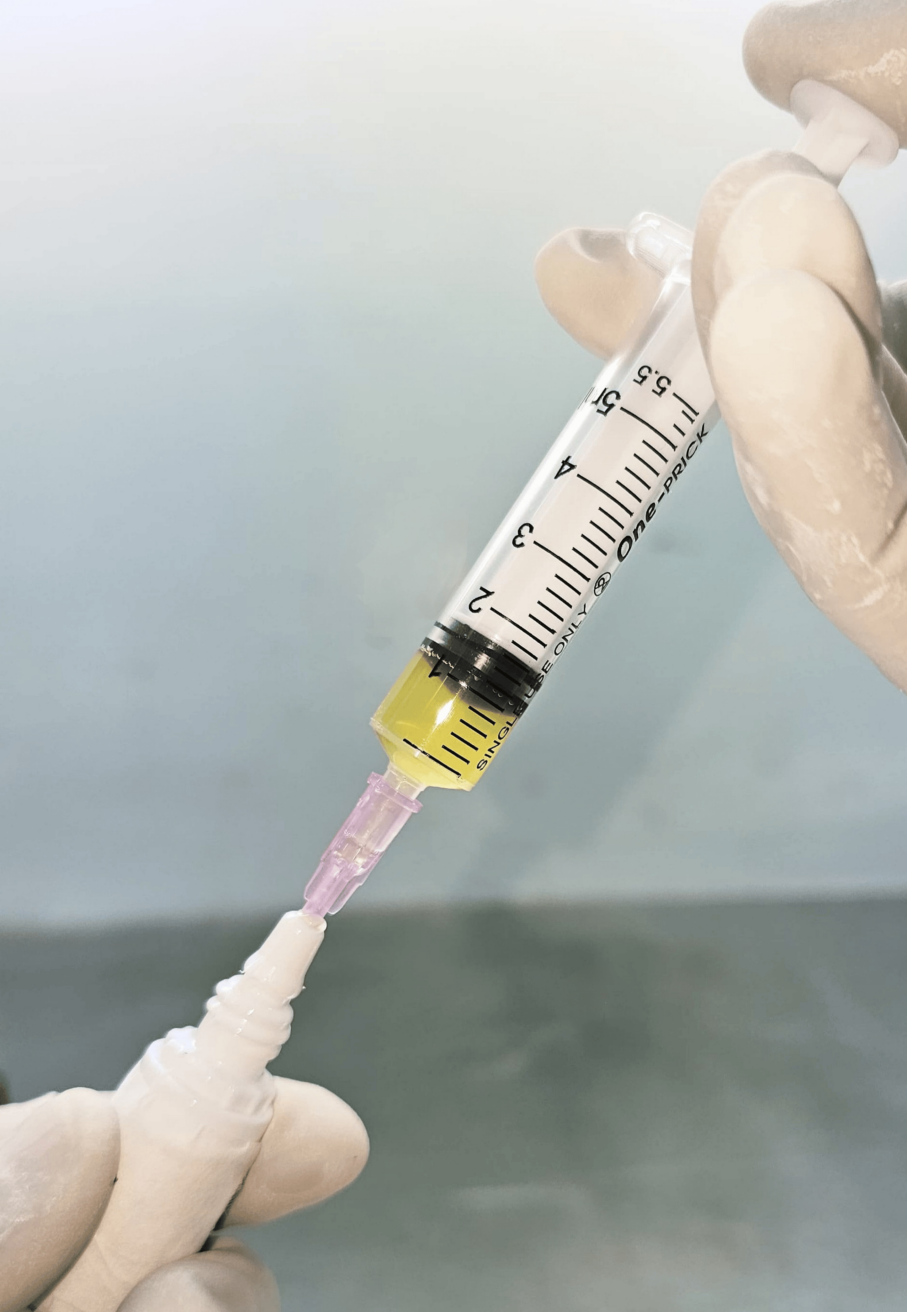
Autologous PRP being transferred to a sterile 5 ml eye-drop bottle under LAF cabin LAF: laminar air flow; PRP: platelet-rich plasma

The entire procedure was performed under sterile conditions. The preparation was done according to the protocol given by Alio et al., who showed that platelet count reaches up to 800,000/microliter in the autologous platelet-rich plasma [13,14]. It is based on a one-step centrifugation technique. Peripheral blood was collected into a 10 ml sterile citrate vial (3.2% sodium citrate, 1 ml) to prevent clotting. It was centrifuged at 1600 rotations per minute (RPM) for 10 minutes at 5°C under optimal conditions. This process gave a plasma fraction enriched with platelets (Figure 1). Under a laminar air flow (LAF) cabin, 3-4 ml plasma aliquots were aspirated (Figure 2). One part was mixed with four parts of balanced salt solution to make a 20% concentration. Next, it was transferred into sterile 5 ml eye-drop bottles (Figure 3). One PRP eye drop bottle was given to the patient, and the rest of the bottles were stored frozen at -20°C in the blood bank. These were later dispensed to patients on their upcoming follow-ups. Patients were instructed to maintain good hygiene: to wash hands, keep the area of application clean, and not touch the tip of the eye-dropper. Patients were instructed to keep the in-use PRP bottle at a temperature of 2 to 4°C. At the end of 3 months, the same procedure was repeated.

To ensure patient compliance with storage, refrigeration, and hygiene instructions at home, we relied entirely upon reporting by the patients. We had no technical tracking mechanism to assess patient adherence and potential chances of PRP degradation over time. Also, since we did not encounter any major adverse effects during our study, we did not feel much stronger reason not to trust the patients’ statements.

Statistical analysis

Data analysis for mean, mean difference (MD), and standard deviation (SD) was done using SPSS Statistics 22 (IBM Corporation) on Microsoft Windows 10. Normality of data was assessed using the Shapiro-Wilk test. Statistical analyses used a paired student’s t-test to compare pre- and post-treatment outcomes within each group (n = 50 patients) and an unpaired student’s t-test to compare post-treatment results between PRP and AT groups (n = 50 patients/group). All parameters were expressed as mean ± standard deviation. Readings from the eyes of the same patient were averaged to ensure independence. The “t” value with the appropriate sign (±) was calculated to indicate the direction of the difference between the compared groups. For all calculations and analyses, a P-value < 0.05 was considered statistically significant.

## Results

The study involved 100 participants diagnosed with chronic evaporative moderate to severe DED in the 18- to 45-year age group. Table 1 presents the demographic data of both groups. Out of 100 patients, 47% were females and 53% were males. The mean age was 32.4 ± 6.2 years and 33.2 ± 5.8 years in the PRP group and AT group, respectively. All patients completed the follow-up period. Table 2 presents within-group analysis through comparison of all parameters in the PRP and AT groups (n = 50 each, MD = post-treatment score - pre-treatment score). Table 3 shows post-treatment between-the-groups analysis (n = 50/group, MD = PRP value - AT value). OSDI and CFS scores showed a significant reduction in both study groups, but the reduction was more in the PRP group, whereas the TBUT score increased significantly only in the PRP group. BCVA score (LogMAR) reduced significantly in the PRP group, but no significant change was found in the AT group. And finally, although ST scores showed improvement in both study groups, it was statistically nonsignificant.

**Table 1:** Patient demographic data PRP: platelet-rich plasma; AT: artificial tears

	PRP Group	AT Group
No. of patients	50	50
Males	26	27
Females	24	23
Mean Age (years)	32.4 ± 6.2	33.2 ± 5.8

**Table 2:** Comparison of pre-treatment and post-treatment parameters in the PRP and AT group The negative MD and t-values indicate improvement for OSDI, CFS, and BCVA (lower scores = better). The positive t-values indicate improvement for TBUT (higher values = better). For Schirmer’s test, the near-zero t-values show no significant change. *significant (P < 0.05); (paired student’s t-test used). PRP: autologous platelet-rich plasma; AT: artificial tears; OSDI: ocular surface disease index; CFS: corneal fluorescein staining; ST: Schirmer’s test; TBUT: tear breakup time; NS: nonsignificant; BCVA: best-corrected visual acuity; SD: standard deviation; MD: mean difference; Pre-T: pre-treatment; Post-T: post-treatment (at 6 months).

Groups	Parameters	Pre-T (Mean ± SD)	Post-T (Mean ± SD)	MD	t-value	P-value
PRP	OSDI score	78.5 ± 17.2	42.7 ± 23.9	-35.8	-12.41	<0.0001*
	CFS score	2.88 ± 0.42	1.19 ± 0.86	-1.69	-15.23	<0.0001*
	ST (mm)	3.89 ± 2.22	3.92 ± 2.80	0.03	0.08	0.94^(NS)^
	TBUT (s)	2.32 ± 2.11	4.26 ± 2.58	1.94	7.32	<0.0001*
	BCVA (LogMAR)	0.22 ± 0.06	0.17 ± 0.05	-0.05	-6.67	<0.0001*
AT	OSDI score	73.7 ± 15.8	63.6 ± 17.2	-10.1	-4.83	<0.0001*
	CFS score	2.21 ± 0.48	2.02 ± 0.8	-0.19	-2.89	0.006*
	ST (mm)	4.26 ± 1.82	4.32 ± 1.84	0.06	0.21	0.83^(NS)^
	TBUT (s)	2.33 ± 1.11	2.98 ± 1.5	0.65	1.72	0.09^(NS)^
	BCVA (LogMAR)	0.20 ± 0.05	0.195 ± 0.05	-0.005	-0.56	0.58^(NS)^

**Table 3:** Comparison of post-treatment parameters between PRP and AT groups at the end of 6 months The negative mean difference and t-values for OSDI, CFS, and BCVA suggest that the PRP group performed better (lower scores = lesser symptoms and disease severity). The positive t-value for TBUT suggests that the PRP group performed better (higher scores = better tear stability). For Schirmer’s test, the near-zero t-value suggests no significant difference between the groups. *significant (P < 0.05), NS: nonsignificant (unpaired student’s t-test used). PRP: autologous platelet-rich plasma; AT: artificial tears; OSDI: ocular surface disease index; CFS: corneal fluorescein staining; ST: Schirmer’s test; TBUT: tear breakup time; BCVA: best-corrected visual acuity; SD: standard deviation; MD: mean difference

Parameters	PRP Group (Mean ± SD)	AT Group (Mean ± SD)	MD	t-value	P-value
OSDI score	42.7 ± 23.9	63.6 ± 17.2	-20.9	-5.12	<0.0001*
CFS score	1.19 ± 0.86	2.02 ± 0.8	-0.83	-5.08	<0.0001*
ST (mm)	3.92 ± 2.8	4.32 ± 1.84	-0.4	-0.85	0.4^(NS)^
TBUT (s)	4.26 ± 2.58	2.98 ± 1.5	1.28	3.08	0.003*
BCVA (LogMAR)	0.17 ± 0.05	0.195 ± 0.05	-0.025	-2.15	0.034*

In Table 2, in the PRP group, the OSDI score significantly decreased from 78.5 ± 17.2 to 42.7 ± 23.9 (MD = -35.8; t = -12.41; P < 0.0001), indicating symptomatic relief. CFS scores also showed a significant reduction (MD = -1.69; t = -15.23; P < 0.0001), showing ocular surface healing. TBUT improved significantly (MD = 1.94; t = 7.32; P < 0.0001), suggesting a positive impact on tear film stability. BCVA showed a modest but statistically significant improvement, whereas ST values showed no significant change (P = 0.94).

In contrast, the AT group in Table 2 showed a smaller but statistically significant decrease in OSDI scores from 73.7 ± 15.8 to 63.6 ± 17.2 (MD = -10.1; t = -4.83; P < 0.0001) and in CFS scores from 2.21 ± 0.48 to 2.02 ± 0.80 (MD = -0.19; t = -2.89; P = 0.006). However, no statistically significant difference was seen in ST (P = 0.83), TBUT (P = 0.092), and BCVA (P = 0.58).

Table 3 presents a post-treatment comparison between the PRP and AT groups. The mean OSDI score was significantly lower in the PRP group (42.7 ± 23.9) compared to the AT group (63.6 ± 17.2), with an MD of -20.9 (t = -5.12; P < 0.0001). The CFS score also showed a statistically significant reduction in the PRP group as compared to the AT group, with an MD of -0.83 (t = -5.08; P < 0.0001). TBUT was significantly higher in the PRP group than in the AT group (P = 0.003). BCVA improved modestly but significantly in the PRP group compared to the AT group (MD = -0.025, P = 0.034). In contrast, the ST did not show any statistically significant difference (P = 0.4).

Apart from some mild discomfort and sticky sensation reported by a few patients, we did not encounter any significant adverse effects throughout our study.

## Discussion

The recent trends, such as prolonged screen time and normalized air-conditioned indoor work environments with low humidity, are negatively impacting the tear film quality. All this is contributing to the rising incidence of DED in recent times. However, the reported prevalence rates vary globally and across India, likely due to factors such as racial differences, different definitions, and different test methods for its diagnosis [15]. Being one of the most prevalent ophthalmic disorders, DED may not only have adverse effects on the quality of life but also impact the results of various ocular surgeries [16].

The management strategy for dry eyes to date mainly relies on symptomatic relief [7,9]. The commercially available topical drugs do relieve dry eye related symptoms; they keep the ocular surface moistened but are devoid of nutrients, and they do not address the underlying cause. Due to decreased efficacy after prolonged treatment, there is a need for more frequent dosing of these drops. Also, anti-inflammatory agents like topical corticosteroids and cyclosporine-A are required in more severe cases [17]. Hemoderivatives are an alternative approach; they have components with properties that promote ocular surface healing, rejuvenation, and immunological protection.

Fox et al. first described the use of AS in keratoconjunctivitis sicca, followed by Tsubota et al. for dry eye with Sjögren’s syndrome [2,4]. However, Urzua et al. and Tananuvat et al. found no statistically significant benefit of AS over artificial tears in their respective studies [18,19]. The presence of vitamins and fibronectin with their epitheliotropic properties, lack of preservatives, its natural tear film-like physical behavior, osmolarity, pH, and biomechanical properties shows its considerable benefits over artificial tears [20,21]. But there are inconsistencies in its preparation, dilution, storage methods, and optimal concentration. Also, since the preparation process of AS results in the elimination of platelets, it results in a significantly lower concentration of growth factors [20].

Plasma rich in growth factors (PRGF), another hemoderivative, is proposed as a new alternative treatment for DED. Experimental findings indicate that PRGF exhibits better anti-inflammatory and proliferative effects compared to AS in a cell culture inflammatory model [11,22].

Similar to PRGF, recently topical PRP is also being investigated as a possible new alternative therapy [11]. This is a preservative-free autologous hemoderivative, and when stored at −20°C, it can maintain its therapeutic efficacy without significant loss of its key growth factors for up to 3 months [23]. PRP contains vitamin A, epidermal growth factor (EGF), nerve growth factor (NGF), platelet factor IV, and insulin-like growth factor 1 (IGF-1) [24]. A key advantage of this product is its high platelet concentration and prolonged growth factor release. Moreover, autologous PRP has shown successful results in many other ocular surface diseases, such as PED, alkali burns, 92% significant to 50% complete resolution in dormant corneal ulcer patients, corneal reconstruction in corneal perforation cases, and 84% improvement in post-LASIK ocular surface syndrome patients [13,25,26].

Our study showed a 45.6% reduction in OSDI score with PRP (MD = -35.8, P < 0.0001) and 13.7% in the AT group (MD = -10.1, P < 0.0001). Post-treatment comparison further confirmed PRP’s better performance (MD = -20.9, P < 0.0001). Yılmaz et al. reported similar results with a 48.4% improvement in dry eye symptoms with PRP [24]. Celebi et al. showed a 55.18% reduction in the AS group compared to 19.5% in the AT group (P < 0.001), reinforcing our results [27]. Similar results were noted by Alio et al., Merayo-Lloves et al., and Wang et al. [28-30].

The CFS score decreased by 58.6% in the PRP group (MD = -1.69, P < 0.0001) and by 8.6% in the AT group (-0.19, P = 0.006). Post-treatment CFS score was also significantly lower in the PRP group (P < 0.0001). Similar results were noted by Alio et al. and Wang et al. [28,30].

For Schirmer’s test, the PRP group and AT group showed improvement by 0.7% and 1.4%, respectively. Although scores increased in both groups, neither PRP (P = 0.94) nor AT (P = 0.834) showed significant improvement. The post-treatment comparison also showed a nonsignificant change (P = 0.4). Celebi et al. and Wang et al. compared AS with AT and reported similar results [27,30].

TBUT significantly improved by 78.01% in the PRP group, whereas the AT group, with a 27.89% improvement, showed no significant change. Post-treatment TBUT remained higher in the PRP group (P = 0.003). This is consistent with the results noted by Yılmaz et al., Celebi et al., and Wang et al. [24,27,30].

For BCVA, a small but statistically significant improvement was observed in the PRP group (MD = -0.05 LogMAR, P < 0.0001), while AT had no significant effect (P = 0.58). Post-treatment BCVA was also better with PRP (P = 0.034). This suggests that PRP’s ocular surface healing may marginally enhance visual function. Our results in the PRP group were consistent with the study done by Alio et al. and Merayo-Lloves et al. [15,29].

These results suggest that PRP has a positive impact on ocular surface healing and tear film stability, as suggested by the CFS score and TBUT score, respectively. It decreases surface inflammation and provides better symptomatic relief, as suggested by the OSDI score. The BCVA score indicates a modest gain in vision. At the same time, ST results suggest that tear quantity (lacrimal gland function) may not change significantly with either treatment.

Strengths

The study provides a holistic understanding of topical autologous PRP implications on the ocular surface for a period of 6 months. The national capital region is our study location, which is one of the most polluted places in the world. The age group of 18 years to 45 years in the inclusion criteria helped us to focus on its impact on the evaporative type of dry eye, since the majority of patients were suffering from computer vision syndrome, along with poor air quality. The comprehensive examination and the above results give a valuable insight into future implications of autologous PRP eye drops as an attractive alternative option for refractory DED, reducing our dependence on long-term use of artificial tears.

Limitations

The single-center study design of the study limits the generalizability of its results. Blinding was not implemented, although many interventional studies comparing visibly distinct treatments face this challenge. Inclusion criteria need to be broadened. A narrow age range limits the generalizability of our study findings. A larger, more diverse sample, including the rural population, is necessary, considering rising screen time and declining air quality across the regions. Subgroup analysis by age, gender, and severity is needed. If logistically feasible in future studies, a hemocytometer can ensure consistency in platelet counts in PRP. In future studies, some patient compliance-ensuring mechanism for refrigeration protocol and temperature monitoring at home is advised. Long-term follow-up is needed to evaluate the sustainability of its effects. Logistical challenges need to be addressed in resource-limited areas.

## Conclusions

We conclude that in chronic evaporative DED patients, autologous PRP showed significantly better clinical outcomes than AT, and being preservative-free due to its autologous origin and without any serious side effects, it was found to be safe when given for a 6-month duration. Added advantages are its ease of preparation and cost-effectiveness.

Our study strengthens the body of evidence shown by previous research found in the Ophthalmology literature. However, standardized international guidelines are needed for its preparation and quality control.
